# Differential Genes Expression between Fertile and Infertile Spermatozoa Revealed by Transcriptome Analysis

**DOI:** 10.1371/journal.pone.0127007

**Published:** 2015-05-14

**Authors:** Sandeep Kumar Bansal, Nishi Gupta, Satya Narayan Sankhwar, Singh Rajender

**Affiliations:** 1 Division of Endocrinology, Central Drug Research Institute, Lucknow, India; 2 Department of Urology, King George’s Medical University (KGMU), Lucknow, India; National University of Singapore, SINGAPORE

## Abstract

**Background:**

It was believed earlier that spermatozoa have no traces of RNA because of loss of most of the cytoplasm. Recent studies have revealed the presence of about 3000 different kinds of mRNAs in ejaculated spermatozoa. However, the correlation of transcriptome profile with infertility remains obscure.

**Methods:**

Total RNA from sperm (after exclusion of somatic cells) of 60 men consisting of individuals with known fertility (n=20), idiopathic infertility (normozoospermic patients, n=20), and asthenozoospermia (n=20) was isolated. After RNA quality check on Bioanalyzer, AffymetrixGeneChip Human Gene 1.0 ST Array was used for expression profiling, which consisted of >30,000 coding transcripts and >11,000 long intergenic non-coding transcripts.

**Results:**

Comparison between all three groups revealed that two thousand and eighty one transcripts were differentially expressed. Analysis of these transcripts showed that some transcripts [ribosomal proteins (*RPS25*, *RPS11*, *RPS13*, *RPL30*, *RPL34*, *RPL27*, *RPS5*), *HINT1*, *HSP90AB1*, *SRSF9*, *EIF4G2*, *ILF2*] were up-regulated in the normozoospermic group, but down-regulated in the asthenozoospermic group in comparison to the control group. Some transcripts were specific to the normozoospermic group (up-regulated: *CAPNS1*, *FAM153C*, *ARF1*, *CFL1*, *RPL19*, *USP22*; down-regulated: *ZNF90*, *SMNDC1*, *c14orf126*, *HNRNPK*), while some were specific to the asthenozoospermic group (up-regulated: *RPL24*, *HNRNPM*, *RPL4*, *PRPF8*, *HTN3*, *RPL11*, *RPL28*, *RPS16*, *SLC25A3*, *C2orf24*, *RHOA*, *GDI2*, *NONO*, *PARK7*; down-regulated: *HNRNPC*, *SMARCAD1*, *RPS24*, *RPS24*, *RPS27A*, *KIFAP3*). A number of differentially expressed transcripts in spermatozoa were related to reproduction (n = 58) and development (n= 210). Some of these transcripts were related to heat shock proteins (*DNAJB4*, *DNAJB14*), testis specific genes (*TCP11*, *TESK1*, *TSPYL1*, *ADAD1*), and Y-chromosome genes (*DAZ1*, *TSPYL1*).

**Conclusion:**

A complex RNA population in spermatozoa consisted of coding and non-coding RNAs. A number of transcripts that participate in a host of cellular processes, including reproduction and development were differentially expressed between fertile and infertile individuals. Differences between comparison groups suggest that sperm RNA has strong potential of acting as markers for fertility evaluation.

## Introduction

Sperm is a small, highly specialized and compact cell that delivers the male genetic material to the next generation. Initially, it was thought that spermatozoa serve the function of only delivering the DNA and have no traces of RNA because of loss of most of the cytoplasm. Grunewald et al (2005) revealed by *in vitro* radio-labeling experiments that mature human spermatozoa are transcriptionally inactive [1]. However, recent studies have shown the presence of RNA in sperm using RT-PCR [2,3], targeted microarrays [4], differential display method [5], and subtractive hybridization experiments [6]. According to a study by Pessot et al (1989), human or rat sperm contain an average 0.1pg of RNA [7]. Previous studies using microarray analysis have revealed the presence of about 3000 different kinds of mRNAs in the ejaculated spermatozoa that is about 0.015 pg of the total RNA [8]. Lalancette et al (2009) revealed that a series of RNA transcripts in human spermatozoa is strictly regulated among a heterogeneous population of RNA transcripts [9].

Studies exploring the complexity of spermatozoal RNA population indicated the presence of rRNA (Ribosomal ribonucleic acid), mRNA (messenger ribonucleic acid), sncRNAs (small non-coding RNAs) and large non-coding RNAs [10,11]. Recently, Krawetz et al (2011) revealed the percentage distribution of sncRNAs in human spermatozoa [11]. According to this study, approximately 7% miRNAs (microRNAs), 11% piRNAs (Piwi-interacting RNAs) and 65% repeat-associated small RNAs are present in human spermatozoa. Recently, some researchers identified novel sncRNAs (spR-12 and -13) present in mouse spermatozoa, zygote, and early embryo that did not correspond to known piRNAs and miRNAs [10]. A study showed the presence of high levels of transcribed repetitive sequences including medium reiteration repeats (MERs) and short and long nuclear interspersed repeats (SINES and LINES) in human spermatozoa [12]. Earlier, Kumar et al (1993) demonstrated the presence of c-myc (a proto-oncogene) mRNA transcript in human spermatozoa using RT-PCR and *in situ* hybridization [13]. Therefore, the presence of RNA in sperm is not dubious.

Now since the presence of RNAs in sperm is ascertained, their functional importance is a matter of debate. The most interesting queries are regarding a probable correlation between RNA profile of sperm and male infertility, and if these RNAs play roles in downstream development post-fertilization. Answers to both these questions require a detailed investigation of sperm RNA content. One theory suggests that mRNAs seen in mature spermatozoa could be the remnants of RNAs synthesized during spermatogenesis. This theory is supported by the fact that mRNAs seen in spermatozoa coincide with those found in human testes [3]. If the suggested theory were true, gene expression levels would decide the remnants of transcripts in spermatozoa. Probably, this would help decide the patient’s fertility status or the fertility potential of sperm. Moreover, the mechanism of selection of the transcripts in sperm is still unknown; however, it may be non-selective. Irrespective of the reasons behind the presence of RNA in sperm, the most intriguing question to investigate is whether we can take advantage of sperm RNA content in understanding the fertility status of an individual.

Further, the fate of RNA transferred to oocyte upon fertilization is under investigations. It is possible that some of the transcripts transferred to oocyte are degraded and the rest would participate in the development of oocyte. Nevertheless, identification of paternal transcripts in oocyte may help decide several features of the next progeny including their health and fertility. Though the identification of RNA content of spermatozoa could reveal a lot about early embryo development, individual phenotype and fertility status, yet very little is known about sperm RNA population, their role in male fertility and correlation with infertility. In view of the above, we hypothesized that any testicular disturbance or imbalance that makes changes in sperm production and consequently fertility may modify spermatozoal RNA profile. In fact, the differences in sperm RNA profile between fertile and infertile men should identify the specific genes and the pathways necessary for the production of fertile spermatozoa. To test this hypothesis, we compared the RNA profiles of spermatozoa obtained from asthenozoospermic, normozoospermic, and fertile individuals.

## Materials and Methods

### Sample collection and processing

The Institutional Review Board and Ethics Committee of the Central Drug Research Institute (CDRI) approved this study. Semen samples were collected from the subjects according to the WHO criteria for semen collection and analysis [14]. All subjects were informed about the purpose of sample collection and informed written consents were obtained. Study subjects were recruited from Department of Urology, King George’s Medical University, Lucknow. Male infertility was defined as the inability to initiate a pregnancy after one year or more of regular unprotected intercourse. Before sample collection, all subjects underwent detailed medical and physical examinations. The possibility of female factors was excluded before enrollment of male partner in the study. Further, the patients with endocrine abnormalities (hypogonadism) and those with gross dysmorphic abnormalities, acquired and congenital structural defects of urogenital system (cystic fibrosis, Young’s syndrome, etc), were excluded. The patients with the history of surgical intervention of genital tract obstruction/dysfunction (varicocele, obstructive azoospermia) were also excluded. Infertile individuals used to excessive alcohol consumption, smoking, drug abuse (ecstasy, marijuana and recreational substances), and those having been exposed to radiations as a part of radiotherapy, were also excluded. Further, the patients showing chromosomal abnormalities or Y-chromosome partial deletions were also excluded. Control samples were taken from the volunteers visiting the clinic for problems other than infertility. All control individuals had fathered at least one child during the last three years and never had any sexual abnormality or infertility. The controls belonged to the same age-group (between 20 and 45 yrs) and had the same ethnicity as that of the patients. Samples were collected from 60 men consisting of individuals with known fertility (n = 20), idiopathic infertility (normozoospermic patients, n = 20), and asthenozoospermia (n = 20). Semen samples were collected after five to seven days of sexual abstinence in a clean, labeled, and wide-mouthed plastic container followed by incubation at 37°C till liquefaction. Ejaculated sperm were purified in two steps to ensure complete absence of somatic cell contamination. In the first step, purified spermatozoa were obtained by using 44 and 88% discontinuous percoll (Sigma Aldrich, USA) centrifugation gradient, as described elsewhere [15]. After a 30-minute gradient centrifugation at 600 g, spermatozoa were recovered from the base of 88% percoll fraction and washed twice with Ham’s F-10 medium containing 10% fetal bovine serum (Sigma Aldrich, USA). In the second step, purified spermatozoa were treated with somatic cell lysis buffer (SCL buffer: 0.1% SDS, 0.5% Triton X in diethylpyrocarbonate (DEPC) treated water) to eliminate the somatic cells. Washed sperm pellet (from the first step) was re-suspended in one ml SCL buffer and incubated on ice for 30 minutes with intermittent mixing. Further, the incubation was extended until microscopic inspection confirmed the absence of somatic cells in the sperm suspension. Finally, the sperm suspension was centrifuged at 220 g for ten minutes at 4°C. Moreover, the purity of spermatogenic cells was confirmed by the failure of amplification of somatic cell markers (E-cadherin; cell junction gene). In each study group, twenty purified and somatic-cell-free sperm samples were pooled and two technical replicates were prepared. Sperm samples were pooled to obtain the amount of RNA (equal to or more than one microgram) required for quality check and array hybridization.

### Sperm RNA isolation, quantification and quality analysis

Total RNA was extracted from sperm using modified TRIzol method. The pooled sperm pellet was suspended in 0.5 mL of TRIzol. The suspension was incubated on a dry bath at 65°C for 10 min and passed through 26 gauge needle using tuberculin syringe until a smooth flow of the suspension was observed. To this suspension, 0.1 ml of chloroform was added. Microtubes containing the suspension were vortexed, incubated at room temperature, and then centrifuged at 12,000 g for 15 min. Following centrifugation, the mixture separates into lower red, phenol-chloroform phase, an interphase, and a colorless upper aqueous phase. RNA remains exclusively in the aqueous phase. The aqueous phase was transferred to a fresh tube carefully, without disturbing the interphase. RNA was precipitated by adding 0.25 mL of chilled isopropyl alcohol. Further, the pellet was washed twice with 0.5 mL of 75% ethanol. The RNA pellet was air-dried for 5–10 min ensuring not to let it dry completely as this may greatly decrease its solubility. The pellet was dissolved in DEPC-treated water by gentle pipetting. The quantity and quality of isolated RNA samples were checked using Agilent Bioanalyzer and by spectrophotometry. RNA quantification was done spectrophotometrically using Nanodrop 2000/2000C. A260/280 ratio (approx. 2.0) for RNA samples calculated by Nanodrop was also considered to assess quality of the RNA preparation. RNA samples were processed immediately for cDNA synthesis, followed by storage at -80°C till further processing.

### Array preparation and hybridization

AffymetrixGeneChip Human Gene 1.0 ST Array (Affymetrix, Santa Clara, CA, USA) was used for expression profiling. These whole-transcript arrays included probes to measure both mRNAs and long intergenic non-coding RNA transcripts (lincRNAs) and provided a complete expression profile for >30,000 mRNAs and >11,000 lincRNAs.

### Array quality control check and preprocessing of the raw data

Affymetrix power tool (APT) was used for quality control check and data normalization prior to the data analysis. The qcc argument to apt-probeset-summarize resulted in the generation of a file containing summary measures for each array, which was assessed to check the quality of the data prior to analysis. Affymetrix array raw data in the form of. CEL file (raw signal intensity data) contained probe-level intensities from a single array (sample). These files were normalized by Affymetrix power tool (APT) using RMA (Robust multi-array average) algorithm, which created an expression matrix from Affymetrix data. By RMA algorithm, raw intensity values were background corrected, log_2_ transformed and then quantile normalized. MA and RMA plots after normalization have been shown in S1 Fig.

Quality assessment of the microarray experiment was done from. CEL files (signal intensity files) obtained after microarray. Quality matrix calculated by Affymetrix power tool was used for quality assessment and microarray experiment performance. All quality control graphs have been shown in S2 Fig. Pm_mean (mean of the raw intensity for all of the PM probes on the array prior to any intensity transformations) value was used to ascertain whether chips were unusually dim or bright. Bgrd_mean value was calculated as a mean of the raw intensity for the probes used to calculate background prior to any intensity transformations. Pos_vs_neg_auc (the area under the curve (AUC) for a receiver operating characteristic (ROC) curve was calculated for comparing signal values for the positive controls to the negative controls assuming that the negative controls are a measure of the false positives and the positive controls are a measure of the true positives. The ROC curve was generated by evaluating how well the probe set signals separate the positive controls from the negative controls. An AUC value of one reflected perfect separation whereas an AUC value of 0.5 showed no separation. Mad_residual_mean (the mean of the absolute deviation of the residuals from the median) was calculated as a measure of how well or poor all of the probes on a given chip fit the model [Different probes (features on the chip) will return different intensities when hybridized to a common target]. Rle_mean [the mean absolute relative log expression (RLE) for all the probesets] was used as a reflection of the biological variability of the replicates. Bac_spike [the set of probesets, which hybridize to the pre-labeled bacterial spike controls (BioB, BioC, BioD, and Cre)] were used in identifying problems with the hybridization and/or chip. Polya_spike [the set of polyadenylated RNA spikes (Lys, Phe, Thr, and Dap)] was used in identifying problems with the target preparation. Apart from this, the array chip also included neg_control (the set of putative intron based probe sets from putative housekeeping genes) and pos_control (the set of putative exon based probe sets from putative housekeeping genes). Nevertheless, the pos_control and all probeset categories were used for estimation of the overall quality of the data from each chip.

### Microarray data analysis and statistical methods

Normalized microarray data were analyzed using the modules available on web-server (www.arraymining.net) [16]. These modules included: gene selection analysis, gene set analysis, and gene co-expression network visualization. Gene selection analysis module used Ensemble feature ranking (which combines the eBayes, SAM, PLS-CV and RF-MDA selection schemes) for gene selection. Gene set analysis module included Parametric Gene Set Enrichment Analysis (PGSEA or PAGE) method by Kim and Volsky (2005) [17], which used a parametric statistical model to identify significantly differentially expressed and functionally related gene sets. Gene co-expression network analysis module included the graph-layout algorithm by Fruchterman and Rheingold. Apart from this, an online tool, PANTHER (Protein Analysis THrough Evolutionary Relationships), was used for gene ontology and pathway analysis [18], and the Database for Annotation, Visualization and Integrated Discovery (DAVID) v6.7 was used for conversion of Affymetrix gene probeset IDs [19]. Gene ontology included assessment of molecular function, biological process, and cellular component analysis of microarray data.

### Three-group comparison

- Gene selection and gene ontology: ArrayMining online tool was used to compare the three study groups together (control, normozoospermic, and asthenozoospermic) for differentially expressed genes (Feature parameter was set at 5000). Subsequently, the gene list thus obtained was fed into PANTHER online tool for gene ontology analysis.
- Gene set analysis: ArrayMining tool was used for gene set analysis, which provided insights into differentially expressed meta-gene set as the pathways affected.
- Co-expression network analysis: ArrayMining tool was used for co-expression network analysis, which provided details of the genes having similar expression as connection components.

### Two-group comparison

- Up- and down-regulated genes: ArrayMining online tool was used to compare two groups (control versus normozoospermic, control versus asthenozoospermic, normozoospermic versus asthenozoospermic) for differentially expressed genes. Feature parameter was set at 100 to identify top 100 ranked differentially expressed probes (genes) in each comparison.
- Gene selection and gene ontology: Further, feature parameter was set at 5000 in the ArrayMining tool to identify maximum differentially expressed probes (genes) in each comparison. Sequentially, the gene list thus obtained was fed into PANTHER online tool for gene ontology analysis.
- Statistical overrepresentation test: PANTHER online tool was used to evaluate the representation of genes in each gene ontology category (cellular localization, biological process, and molecular function) for each comparison.

## Results

### Three-group comparison

#### Gene selection and gene ontology

**Fig 1:**
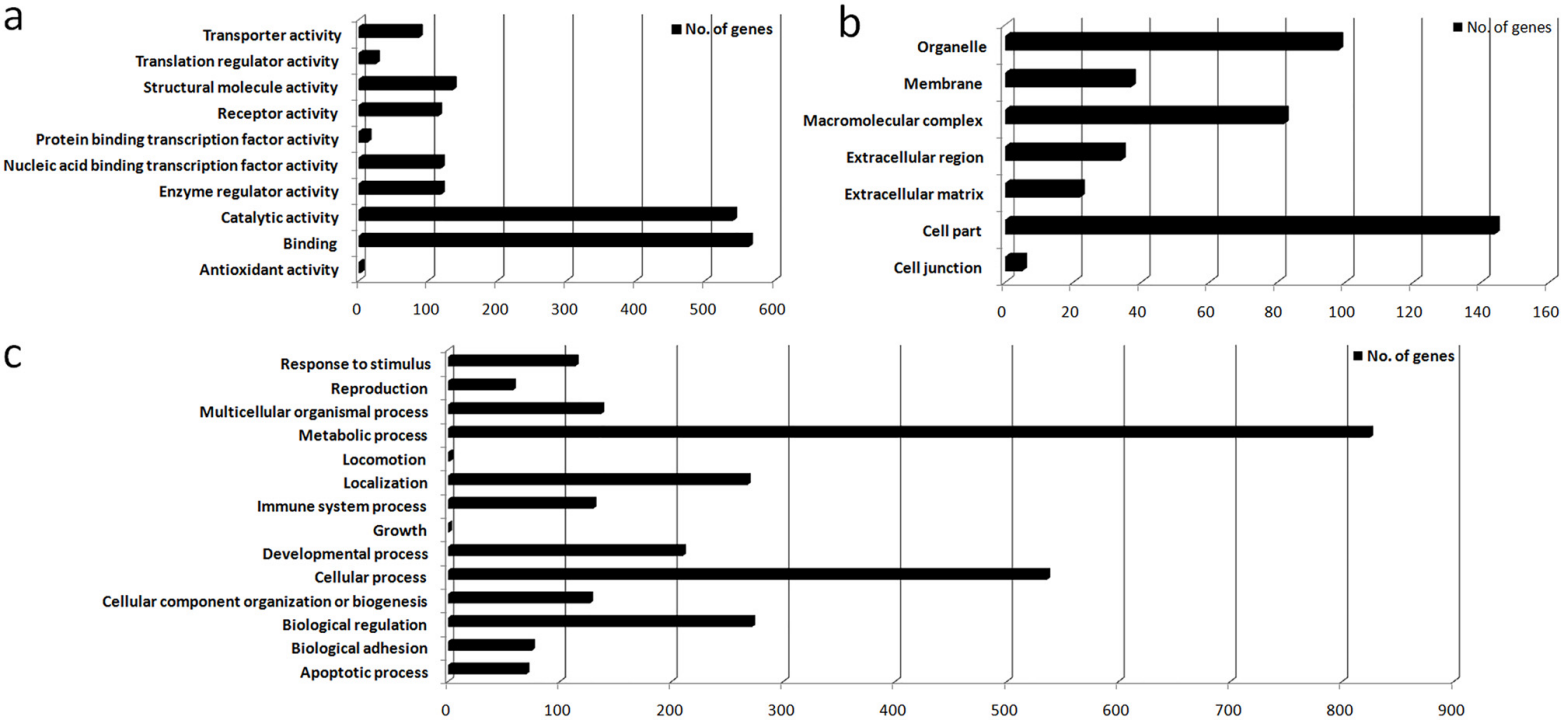
Three group comparison (feature set = 5000) and gene ontology analysis. Bar graph showing gene ontology analysis on the basis of a) molecular function b) cellular location, and c) biological processes, when three groups (fertile control, asthenozoospermic and normozoospermic infertile) were compared simultaneously.

Comparison of all the three groups together revealed that two thousand and eighty one transcripts (feature parameter was set at 5000 and unannotated and duplicate probes were removed) were differentially expressed (S1 Table). Transcriptome profiles of the three groups (controls, asthenozoospermic and normozoospermic) have been shown as heat map in S3 Fig. Differential expression of the top four ranked probes (genes) in the three groups has been shown as box plot in S4 Fig. Analysis of transcripts (using PANTHER) showed that differentially expressed RNAs cover diverse gene sequences localized all round the cell, including organelles, membrane, extracellular matrix, and cell junctions (Fig 1). Various numbers of transcripts had catalytic (540), binding (563), structural molecule (135), enzyme regulator (118), and transcription factor (118) activities. The transcripts were related to various biological processes, but a large number of them were related to metabolic processes (825), cellular processes (536), biological regulation (272), and localization (268).

**Fig 2:**
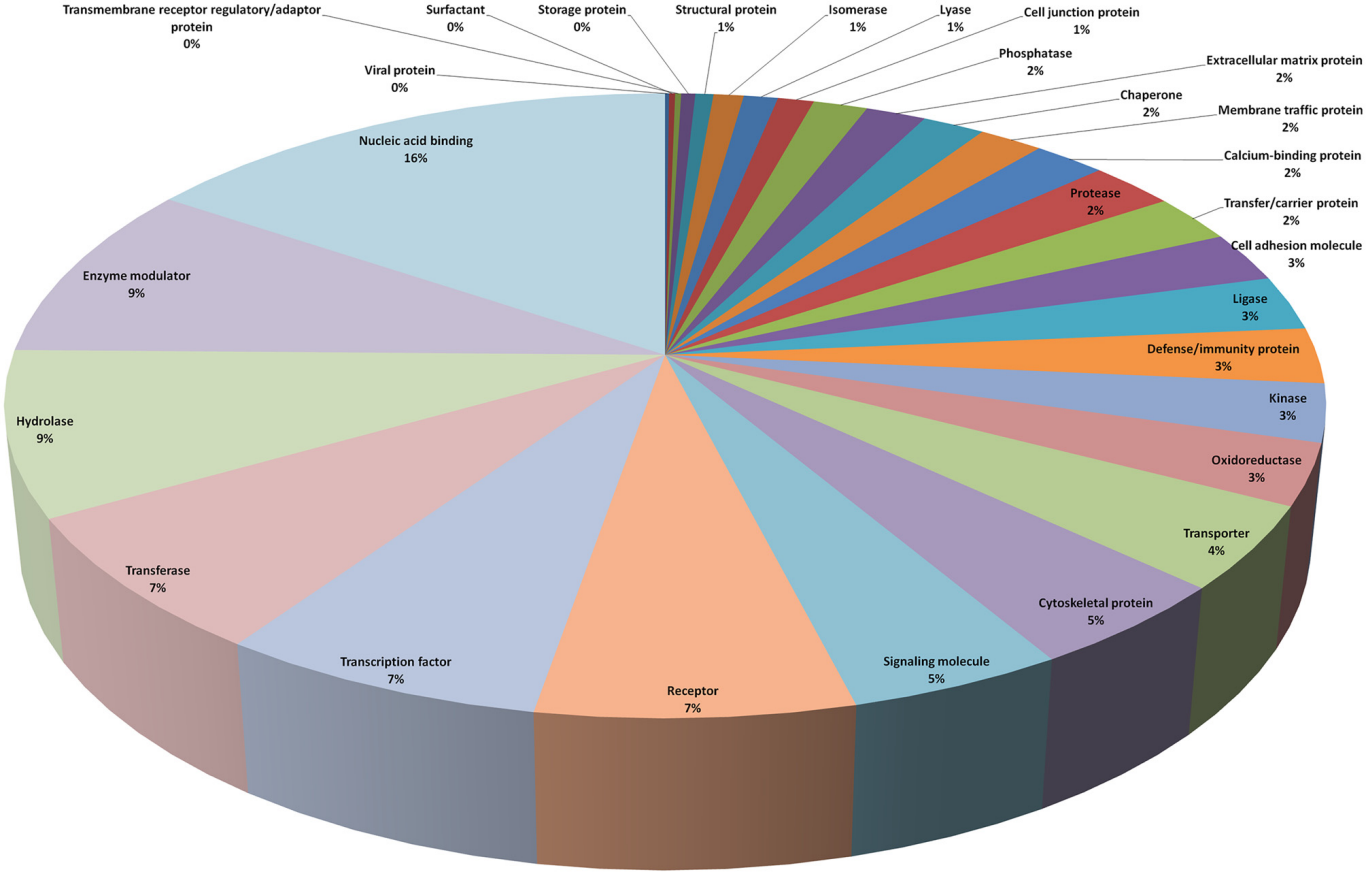
Three group comparison (feature set = 5000) and protein class analysis. PANTHER analysis of most significantly (top 5000) differentially expressed transcripts to classify them into protein classes.

Maximum numbers of transcripts belonged to protein classes of nucleic acid binding (271), enzyme modulators (159), hydrolases (148), transferases (124), transcription factors (120), receptors (116), and signaling molecules (79) (Fig 2). Further, a number of transcripts were related to reproduction (58) and development (210). Some of these transcripts were related to heat shock proteins (*DNAJB4*, *DNAJB14*), testis specific genes (*TCP11*, *TESK1*, *TSPYL1*, *ADAD1*), Y-chromosome genes (*DAZ1*, *TSPYL1*), trans-membrane proteases (*TMPRSS2*, *TMPRSS11F*, *TMPRSS11A*, *TMPRSS11E*), peptidases (*USP22*, *USP34*, *USP15*, *USP25*, *ADAM23*, *ADAM32*, *ADAM2*, *ADAM30*), kinases (*TESK1*, *TXK*, *VRK2*, *MAPKAPK2*, *RIPK2*, *JAK1*) and others (*LPHN3*, *MSH2*, *TSPAN6*, *SEMG2*, *BAI3*, *CRISP2*, *DYNLL1*, *GPR125*, *CCNE2*, *PIWIL1*, *BMP5*, *BMP8B*, *SEMG1*, *PLCZ1*, *BAG5*, *CDH10*, *CHL1*, *DYNC1I2*, *EPHA3*, *KIF23*, *RABGAP1*, *SLC4A7*, *DNAH14*, *MYBL1*, *UBE2D1*, *CCNE2*, *EIF3F*, *PSMD14*).

#### Gene set analysis

Gene set analysis module combined a set of functionally similar genes as a meta-gene set that was used for functional data annotation from the GO database to plot the heat map. The expression levels of all genes in the meta-gene set were analyzed to evaluate the involvement of a pathway in spermatogenesis and fertility. Heat map of gene set analysis has been shown in S5 Fig. Differential expression of the top four ranked pathways has been shown as box plot in S6 Fig. The results of gene set analysis have been tabulated in S2 Table.

**Fig 3:**
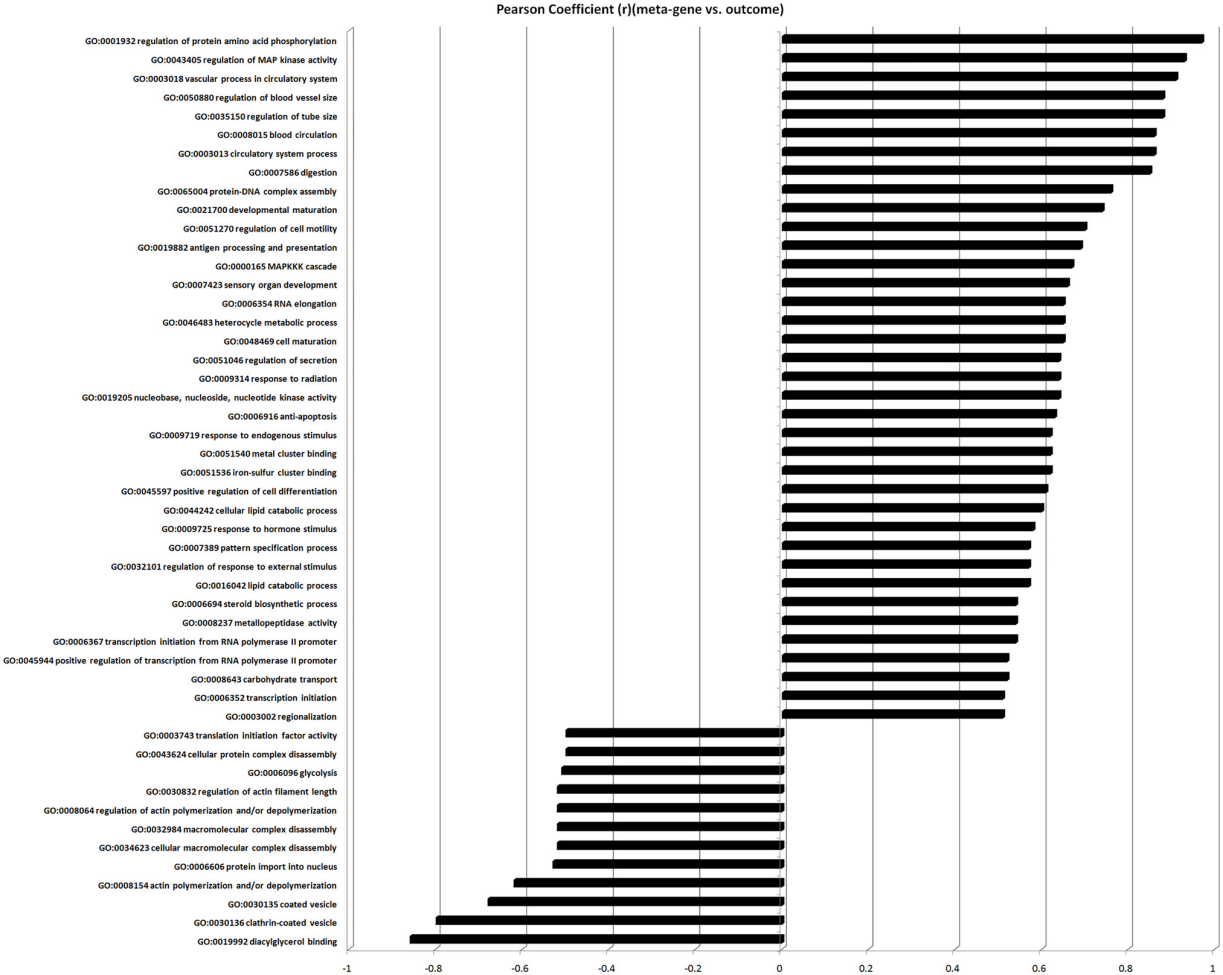
Three group comparison and gene set analysis. Bar graph showing correlation between expression of meta-gene set and the pathway (outcome) involved.

The pathways that were strongly correlated with low expression levels of genes in meta-gene set included: diacylglycerol binding pathway, clathrin-coated vesicle, coated vesicle, actin polymerization and/or depolymerization, protein import into nucleus, cellular macromolecular complex disassembly, macromolecular complex disassembly, regulation of actin polymerization and/or depolymerization, regulation of actin filament length, glycolysis, cellular protein complex disassembly, and translation initiation factor activity (Table 1 and Fig 3).

**Table 1:** Three group comparison and gene set analysis for comparison among normal fertile control, asthenozoospermic infertile, and idiopathic normozoospermic infertile groups.

Pathway name	Pearson coefficient (r) (meta-gene vs. outcome)	Q-values	F-score
**GO:0019992:Diacylglycerol binding**	-0.86	2.50E-02	8.6
**GO:0030136:Clathrin-coated vesicle**	-0.8	4.90E-02	6.06
**GO:0030135: Coated vesicle**	-0.68	4.20E-02	6.6
**GO:0008154:Actin polymerization and/or depolymerization**	-0.62	1.50E-02	10.91
**GO:0006606:Protein import into nucleus**	-0.53	3.50E-02	7.27
**GO:0034623:Cellular macromolecular complex disassembly**	-0.52	3.30E-03	20.97
**GO:0032984:Macromolecular complex disassembly**	-0.52	6.30E-03	15.77
**GO:0008064: Regulation of actin polymerization and/or depolymerization**	-0.52	3.30E-02	7.44
**GO:0030832:Regulation of actin filament length**	-0.52	4.60E-02	6.28
**GO:0006096:Glycolysis**	-0.51	3.30E-03	20.82
**GO:0043624:Cellular protein complex disassembly**	-0.5	3.20E-03	21.34
**GO:0003743:Translation initiation factor activity**	-0.5	3.50E-02	7.18
**GO:0003002:Regionalization**	0.51	1.80E-02	10.04
**GO:0006352:Transcription initiation**	0.51	5.00E-02	5.99
**GO:0008643:Carbohydrate transport**	0.52	8.40E-03	14.02
**GO:0045944: Positive regulation of transcription from RNA polymerase II promoter**	0.52	4.70E-02	6.21
**GO:0006367:Transcription initiation from RNA polymerase II promoter**	0.54	1.80E-02	10.05
**GO:0008237:Metallopeptidase activity**	0.54	2.80E-02	8.06
**GO:0006694:Steroid biosynthetic process**	0.54	3.30E-02	7.47
**GO:0016042:Lipid catabolic process**	0.57	1.10E-02	12.4
**GO:0032101:Regulation of response to external stimulus**	0.57	2.80E-02	8.11
**GO:0007389:Pattern specification process**	0.57	3.40E-02	7.36
**GO:0009725:Response to hormone stimulus**	0.58	1.90E-02	9.76
**GO:0044242:Cellular lipid catabolic process**	0.6	2.20E-02	9.17
**GO:0045597:Positive regulation of cell differentiation**	0.61	3.00E-02	7.85
**GO:0051536:Iron-sulfur cluster binding**	0.62	1.10E-02	12.76
**GO:0051540:Metal cluster binding**	0.62	1.10E-02	12.76
**GO:0009719:Response to endogenous stimulus**	0.62	1.90E-02	9.73
**GO:0006916:Anti-apoptosis**	0.63	2.80E-02	8.08
**GO:0019205:Nucleobase, nucleoside, nucleotide kinase activity**	0.64	6.00E-03	15.97
**GO:0009314:Response to radiation**	0.64	3.10E-02	7.68
**GO:0051046:Regulation of secretion**	0.64	3.80E-02	6.92
**GO:0048469:Cell maturation**	0.65	2.30E-02	8.87
**GO:0046483:Heterocycle metabolic process**	0.65	2.30E-02	8.82
**GO:0006354: RNA elongation**	0.65	2.90E-02	7.95
**GO:0007423:Sensory organ development**	0.66	1.50E-02	11
**GO:0000165: MAPKKK cascade**	0.67	4.70E-03	17.89
**GO:0019882:Antigen processing and presentation**	0.69	4.80E-02	6.13
**GO:0051270:Regulation of cell motility**	0.7	2.70E-02	8.26
**GO:0021700:Developmental maturation**	0.74	3.00E-02	7.8
**GO:0065004:Protein-DNA complex assembly**	0.76	3.40E-02	7.36
**GO:0007586:Digestion**	0.85	1.40E-02	11.12
**GO:0003013:Circulatory system process**	0.86	4.10E-02	6.71
**GO:0008015:Blood circulation**	0.86	4.10E-02	6.71
**GO:0035150:Regulation of tube size**	0.88	1.30E-02	11.8
**GO:0050880:Regulation of blood vessel size**	0.88	1.30E-02	11.8
**GO:0003018:Vascular process in circulatory system**	0.91	1.10E-02	12.91
**GO:0043405:Regulation of MAP kinase activity**	0.93	3.70E-02	6.97
**GO:0001932:Regulation of protein amino acid phosphorylation**	0.97	4.90E-02	6.07

Only pathways that strongly correlated (r≥0.5) with expression of meta-gene set have been shown.

The pathways that were strongly correlated with high expression levels of genes in meta-gene set included: regulation of protein amino acid phosphorylation, regulation of MAP kinase activity, vascular process in circulatory system, regulation of blood vessel size, regulation of tube size, blood circulation, circulatory system process, digestion, protein-DNA complex assembly, developmental maturation, regulation of cell motility, antigen processing and presentation, MAPKKK cascade, sensory organ development, RNA elongation, heterocycle metabolic process, cell maturation, regulation of secretion, and others (Table 1 and Fig 3).

#### Gene co-expression network analysis

**Fig 4:**
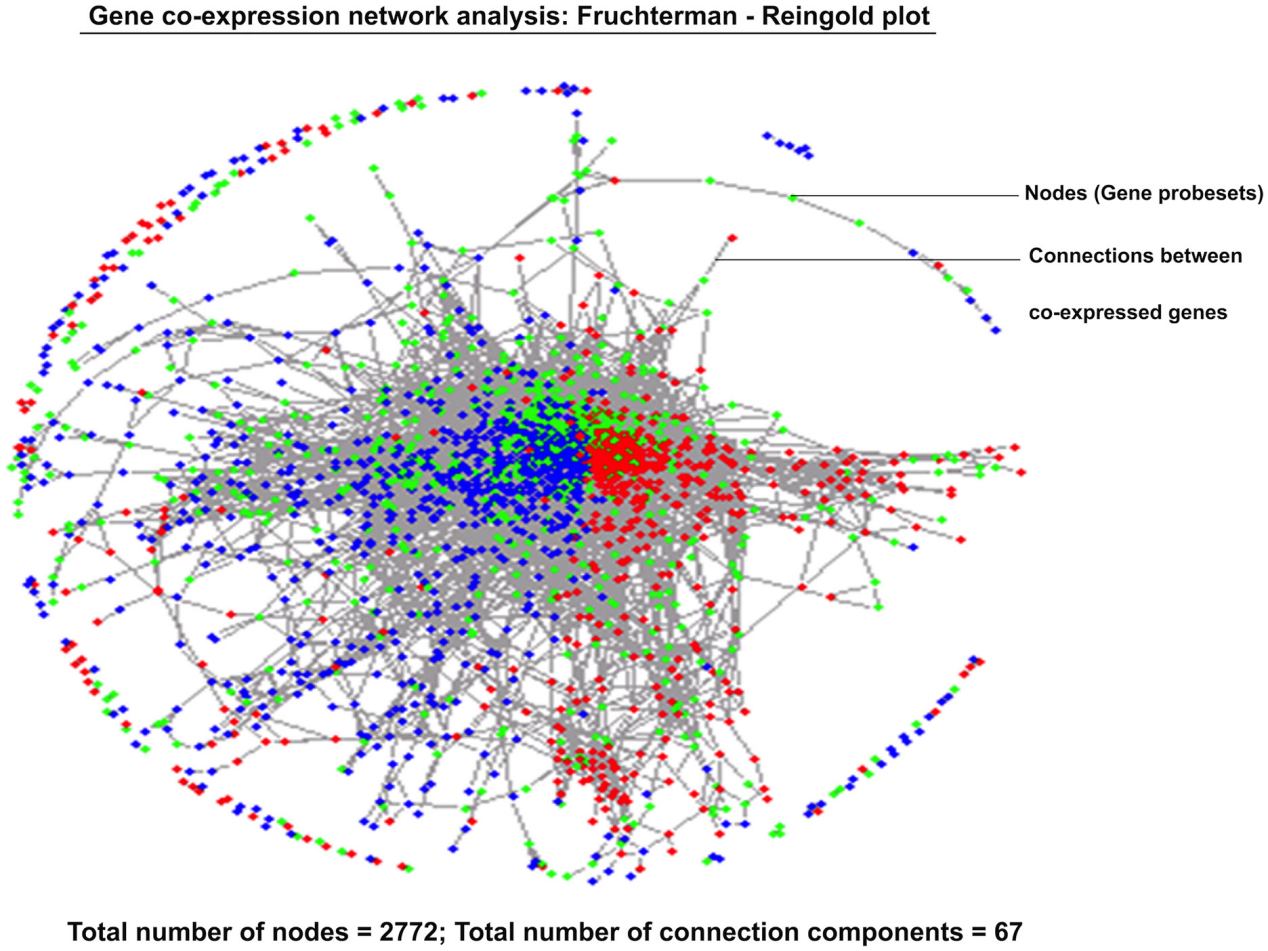
Three group comparison and co-expression network analysis. Fruchterman-Reingold plot showing connections between co-expressing genes. Sample classes are colour-coded and probes are coloured according to the class with the highest median expression value across the corresponding samples. Colour-codes: class 1 (red: asthenozoospermic), class 2 (green: fertile control), and class 3 (blue: normozoospermic infertile).

Co-expressing genes have been shown as Fruchterman-Reingold (FR) plot where nodes represent the Affymetrix probes and edges connect the co-expressed probes (Fig 4). Statistical analysis revealed 2772 and 155011 number of nodes and edges, respectively, in the plot. In the FR plot, sample classes are colour-coded and probes are coloured according to the class with the highest median expression value across the corresponding sample class. Colour codes are: class 1 (red: asthenozoospermic), class 2 (green: fertile control), and class 3 (blue: normozoospermic infertile). The total number of connections established between probes based on their expression levels was sixty-seven. These connection components having probes were further converted into official gene symbols using DAVID gene ID conversion tool. Co-expression gene list with connection components has been given in S3 Table. Co-expression significant criterion was set to 0.5 as edge adjacency threshold or minimum expression threshold. A large number of ribosomal RNA (including 18S and 28S rRNA), ribosomal protein transcripts, small nuclear RNA, and small nucleolar RNA had expression more than set edge adjacency threshold. Several transcripts crossed the edge adjacency threshold and had similar expression (co-expression). Co-expression profile identified new targets having similar expression. Some transcript groups that co-expressed are: *CT47A* members (cancer/testis antigen family 47) and *ANAPC5* (anaphase promoting complex subunit 5); *ARF1* (ADP-ribosylation factor-1) and *CDK11* (cyclin-dependent kinase 11); *DDX5* (DEAD box polypeptide 5), *CANX* (calnexin) and *KARS* (lysyl-tRNA-synthetase); *HSP90* (heat shock protein family 90kda) and *STARD7* (StAR-related lipid transfer domain protein 7); *ILF2* (interleukin enhancer binding factor-2) and *STRC* (stereocilin); *DDX11* (DEAD box polypeptide-11) and *SART1* (squamous cell carcinoma antigen recognized by T-cells); *APOC3* (Apolipoprotein C-III), *OCM2* (oncomodulin-2) and *FAM90A1* (family with sequence similarity 90).

### Two-group comparison

#### Up- and down-regulated genes

**Fig 5:**
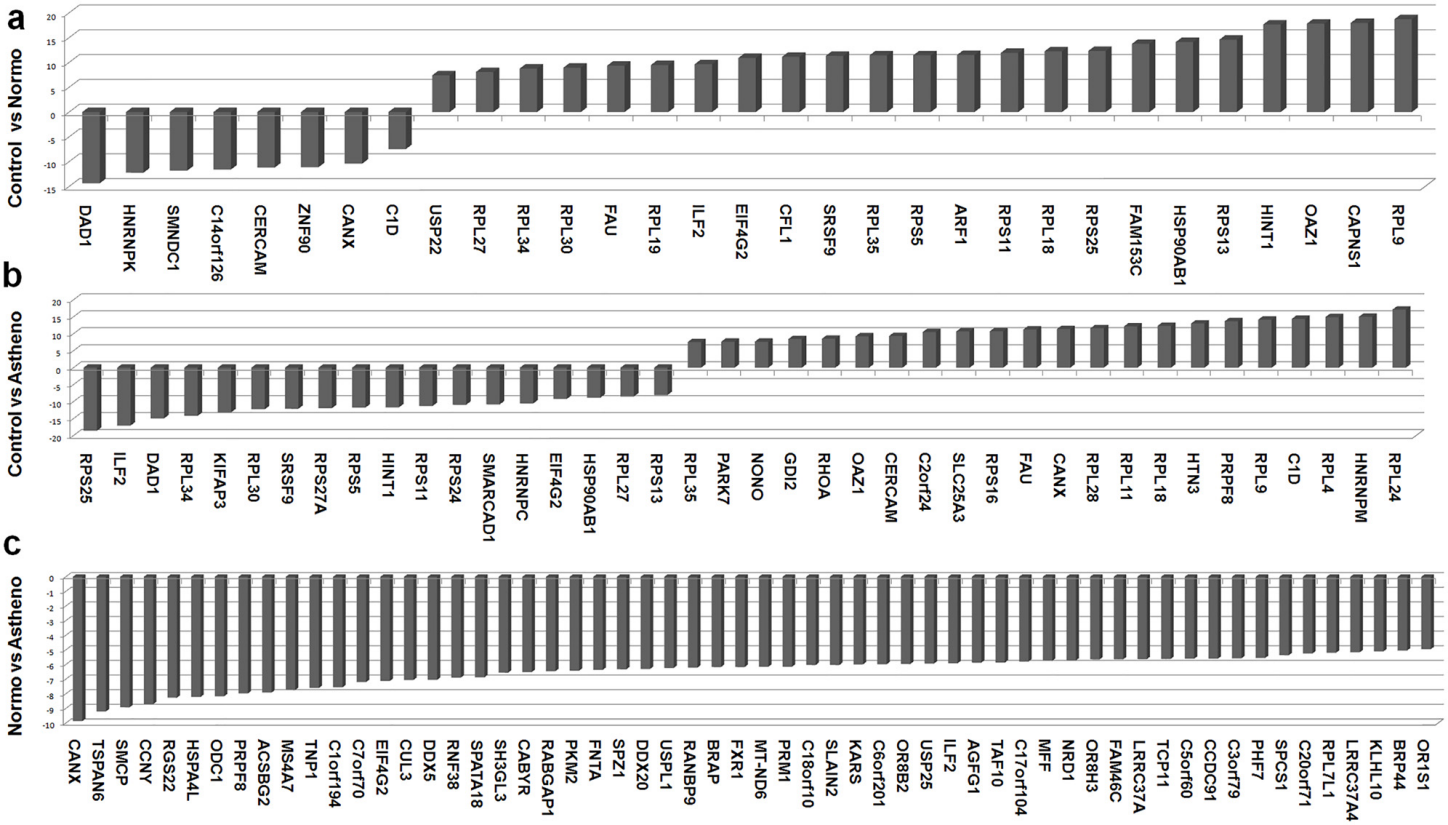
Two group comparison (feature set = 100) and up- and down- regulated genes. Bar graph shows up- and down-regulated genes when the three groups were compared with each other.

Two-group comparison for the top 100 ranked differentially expressed probes (genes) has been shown in Fig 5. Comparison of the asthenozoospermic group with the control group revealed various transcripts to be differentially expressed [**up-regulated**: ribosomal proteins (*RPL24*, *RPL4*, *RPL9*, *RPL18*, *RPL11*, *RPL28*, *RPL35* and *RPS16*), calnexin (*CANX*), *NONO* (non-POU domain containing, octamer-binding), *RHOA* (ras homolog family member A), *OAZ1* (ornithine decarboxylase antizyme 1), *FAU*, *SLC25A3*, *HNRNPM*, *C1D*, *PRPF8*, *HTN3*, *CERCAM*, *GDI2*, *PARK7*, and **down-regulated:** ribosomal proteins (*RPS13*, *RPL27*, *RPS24*, *RPS11*, *RPS5*, *RPS27A*, *RPL30*, *RPL34*, *RPS25)*, *DAD1* (defender against cell death 1), *ILF2* (interleukin enhancer binding factor 2), *SRSF9* (serine/arginine-rich splicing factor 9), *HSP90AB1*, *EIF4G2*, *HNRNPC*, *SMARCAD1*, *HINT1*, *KIFAP3*].

Some transcripts [ribosomal proteins (*RPS25*, *RPS11*, *RPS13*, *RPL30*, *RPL34*, *RPL27*, *RPS5)*, *HINT1*, *HSP90AB1*, *SRSF9*, *EIF4G2*, *ILF2*] were up-regulated in the normozoospermic group, but down-regulated in the asthenozoospermic group when each group was individually compared with the control group. Some transcripts [*RPL9*, *OAZ1*, *RPL18*, *RPL35*, and *FAU*] were up-regulated in both the normozoospermic and asthenozoospermic groups when each was compared with the control group, while only DAD1 transcript was down-regulated in both. Some transcripts were specific to the normozoospermic group (up-regulated: *CAPNS1*, *FAM153C*, *ARF1*, *CFL1*, *RPL19*, *USP22*; down-regulated: *ZNF90*, *SMNDC1*, *c14orf126*, *HNRNPK*), while others were specific to the asthenozoospermic group (up-regulated: *RPL24*, *HNRNPM*, *RPL4*, *PRPF8*, *HTN3*, *RPL11*, *RPL28*, *RPS16*, *SLC25A3*, *C2orf24*, *RHOA*, *GDI2*, *NONO*, *PARK7*; down-regulated: *HNRNPC*, *SMARCAD1*, *RPS24*, *RPS24*, *RPS27A*, *KIFAP3*). In comparison to controls, there was no down-regulation of ribosomal protein transcripts in the normozoospermic group while a number of ribosomal protein transcripts (both from small and large subunits) were down-regulated in the asthenozoospermic group (Fig 5). The number of ribosomal protein transcripts up-regulated was higher in asthenozoospermics in comparison to normozoospermics, when each group was compared with controls.

Comparison of the asthenozoospermic infertile group with the normozoospermic infertile group revealed several transcripts to be differentially expressed. Interestingly, all ranked transcripts were down-regulated when feature selection parameter was set at 100. These transcripts related to various protein classes such as calcium binding protein (*CANX*), cell adhesion molecule (*TSPAN6*), heat shock protein (*HSPA4L*), cytoskeleton protein (*SH3GL3*), immunity protein (*ILF2*), enzyme modulators (*RABGAP1*, *ILF2*, *RGS22*, *AGFG1*), receptor extracellular matrix protein (*LRRC37A*), proteases (*RABGAP1*, *NRD1*, *USP25*), ligases (*KARS*, *ACSBG2*, *RNF38*, *USP25*, *CUL3*), nucleic acid binding proteins (*DDX5*, *KARS*, *PRPF8*, *FXR1*, *PHF7*, *EIF4G2*, *AGFG1*, *RPL7L1*), receptors (*MS4A7*, *TSPAN6*, *TCP11*, *LRRC37A*), signaling molecules (*TSPAN6*, *SPZ1*, *SPCS1*), transcription factor (*TAF10*), transporter proteins (*RANBP9*, *C1orf194*), and transferase (*FNTA*).

#### Gene selection and gene ontology

**Fig 6:**
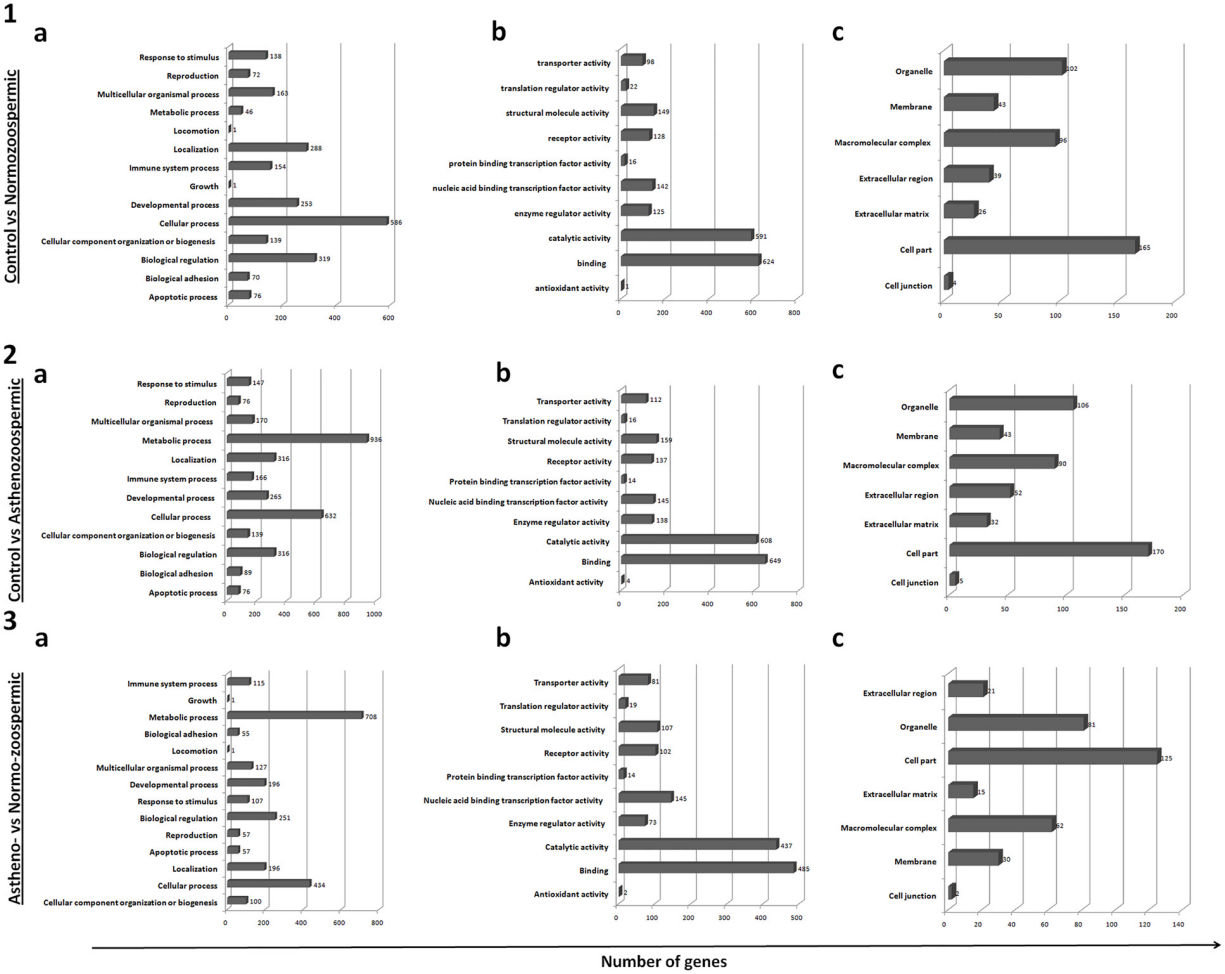
Two group comparison (feature set = 5000) and gene ontology analysis. Bar graph showing gene ontology analysis on the basis of a) biological processes b) molecular function, and c) cellular location when the three groups were compared with each other.

Two-group comparison revealed differential expression of a number of transcripts (control versus normozoospermic = 1895; control versus asthenozoospermic = 2092; normozoospermic versus asthenozoospermic = 1592) (S4 Table). Gene ontology analysis revealed significant variation in the number of differentially expressed transcripts across three comparisons (Fig 6). In comparison to control, maximum number of transcripts in the normozoospermic group were related to cellular processes (n = 586) while those in the asthenozoospermic group were related to metabolic processes (n = 936). The number of transcripts involved in metabolic processes was very low in the normozoospermic group (n = 46), but very high in the asthenozoospermic group (n = 936) in comparison to the control group. Distribution of differentially expressed transcripts in various gene ontology categories [cellular components, molecular function and biological processes (except metabolic and cellular processes)] did not differ significantly (1–15 number of genes) between the normozoospermic and asthenozoospermic groups when each was compared with the control group (Fig 6).

#### Statistical over-representation test

In comparison of the normozoospermic and asthenozoospermic groups with controls, transcripts related to various molecular functions (structural constituent of ribosome, nucleic acid binding, RNA binding, mRNA binding, catalytic activity, hydrolase activity, enzyme regulator activity and DNA helicase activity) were significantly over-represented (Table 2). Further, transcripts related to various biological processes (translation, cell cycle, protein transport, meiosis, chromosome segregation, DNA repair, RNA splicing, mitosis) were significantly over-represented in both the normozoospermic and asthenozoospermic groups in comparison to controls (Table 2). The normozoospermic group saw significant over-representation of transcripts related to vesicle-mediated transport while asthenozoospermic saw over-representation of transcripts involved in fertilization.

**Table 2:** Two-group analysis and statistical over-representation test.

	Gene ontology	Homo sapiens—REFLIST (21804)	No of gene hit	Expected no. of gene hits	Under(-) or over (+)representation of genes	P-value
**Control vsnormozoospermic (Total mapped gene = 1797, unmapped = 98)**	**Molecular Function**					
	Structural constituent of ribosome	204	56	16.81	+	4.420E-12
	Catalytic activity	5529	590	455.68	+	1.340E-10
	Binding	5933	622	488.97	+	5.590E-10
	Nucleic acid binding	3315	360	273.21	+	3.730E-06
	RNA binding	639	94	52.66	+	1.750E-05
	Hydrolase activity	2332	254	192.19	+	5.480E-04
	Structural molecule activity	1261	149	103.93	+	1.610E-03
	Enzyme regulator activity	1091	125	89.92	+	2.920E-02
	mRNA binding	313	46	25.8	+	2.950E-02
	DNA helicase activity	74	17	6.1	+	3.230E-02
	**Biological process**					
	Metabolic process	8613	900	709.85	+	1.26E-17
	Primary metabolic process	7177	738	591.5	+	5.19E-11
	Translation	410	77	33.79	+	1.37E-08
	Protein metabolic process	2807	327	231.34	+	1.58E-08
	Cell cycle	1399	184	115.3	+	9.84E-08
	Protein transport	1339	170	110.36	+	5.06E-06
	Intracellular protein transport	1322	167	108.95	+	9.34E-06
	Meiosis	100	27	8.24	+	2.93E-05
	Cellular process	5952	585	490.54	+	8.84E-05
	Localization	2636	288	217.25	+	9.08E-05
	DNA metabolic process	434	68	35.77	+	1.36E-04
	Transport	2564	279	211.31	+	1.97E-04
	Cellular component organization or biogenesis	1137	139	93.71	+	6.93E-04
	Chromosome segregation	181	35	14.92	+	1.02E-03
	DNA repair	191	35	15.74	+	3.07E-03
	Nucleobase-containing compound metabolic process	3532	357	291.09	+	3.95E-03
	RNA splicing	272	43	22.42	+	1.12E-02
	RNA splicing, via transesterification reactions	272	43	22.42	+	1.12E-02
	DNA replication	221	37	18.21	+	1.15E-02
	Cellular component organization	1065	125	87.77	+	1.23E-02
	mRNA splicing, via spliceosome	368	53	30.33	+	1.83E-02
	Mitosis	528	70	43.52	+	1.91E-02
	Oxidative phosphorylation	57	15	4.7	+	1.99E-02
	Vesicle-mediated transport	928	110	76.48	+	2.30E-02
	Regulation of catalytic activity	1119	128	92.22	+	2.90E-02
	Regulation of molecular function	1140	130	93.95	+	2.92E-02
	**Cellular component**					
	Macromolecular complex	648	96	53.41	+	3.01E-06
	Ribonucleoproteincomplex	134	28	11.04	+	6.36E-04
	Microtubule	248	41	20.44	+	1.84E-03
	Protein complex	514	68	42.36	+	7.36E-03
	**Pathway**					
	No significant over/under representation of genes					
**Control vsasthenozoospermic (total gene mapped = 1979, unmapped = 110)**	**Molecular Function**					
	Structural constituent of ribosome	204	56	18.52	+	2.01E-10
	Binding	5933	647	538.5	+	6.72E-06
	Catalytic activity	5529	607	501.83	+	9.12E-06
	RNA binding	639	98	58	+	1.10E-04
	Nucleic acid binding	3315	373	300.88	+	1.03E-03
	mRNA binding	313	54	28.41	+	1.65E-03
	Structural molecule activity	1261	159	114.45	+	4.33E-03
	Hydrolase activity	2332	267	211.66	+	8.91E-03
	DNA helicase activity	74	19	6.72	+	1.17E-02
	Enzyme regulator activity	1091	138	99.02	+	1.28E-02
	**Biological process**					
	Metabolic process	8613	935	781.74	+	2.93E-10
	Protein metabolic process	2807	351	254.77	+	7.34E-08
	Primary metabolic process	7177	779	651.41	+	1.79E-07
	Translation	410	78	37.21	+	4.24E-07
	Cell cycle	1399	194	126.98	+	1.02E-06
	Localization	2636	317	239.25	+	2.71E-05
	Protein transport	1339	178	121.53	+	6.98E-05
	Intracellular protein transport	1322	175	119.99	+	1.14E-04
	Transport	2564	304	232.72	+	1.73E-04
	Chromosome segregation	181	39	16.43	+	2.38E-04
	Meiosis	100	26	9.08	+	5.56E-04
	Cellular process	5952	631	540.22	+	6.33E-04
	DNA repair	191	39	17.34	+	8.19E-04
	DNA metabolic process	434	70	39.39	+	9.38E-04
	Mitosis	528	81	47.92	+	1.09E-03
	RNA splicing	272	49	24.69	+	1.55E-03
	RNA splicing, via trans-esterification reactions	272	49	24.69	+	1.55E-03
	mRNA splicing, via spliceosome	368	60	33.4	+	3.15E-03
	Regulation of catalytic activity	1119	139	101.56	+	2.90E-02
	Cellular component biogenesis	114	24	10.35	+	3.29E-02
	mRNA processing	438	64	39.75	+	3.67E-02
	Fertilization	82	19	7.44	+	4.68E-02
	Cellular component organization or biogenesis	1137	139	103.2	+	5.58E-02
	**Cellular component**					
	Ribonucleoprotein complex	134	28	12.16	+	3.34E-03
	Macromolecular complex	648	90	58.81	+	3.73E-03
	Cytosol	25	10	2.27	+	6.50E-03
	Ribosome	11	6	1	+	2.99E-02
	Microtubule	248	39	22.51	+	4.77E-02
	**Pathway**					
	No significant over/under representation of genes					
**Asthenovsnormozoospermic (Total gene mapped = 1515, unmapped gene = 75)**	**Molecular Function**					
	Nucleic acid binding	3315	308	230.34	+	8.73E-06
	RNA binding	639	80	44.4	+	1.00E-04
	Structural constituent of ribosome	204	34	14.17	+	7.73E-04
	Binding	5933	486	412.24	+	2.49E-03
	**Biological process**					
	Primary metabolic process	7177	606	498.68	+	7.74E-07
	Metabolic process	8613	706	598.45	+	2.11E-06
	Translation	410	61	28.49	+	1.02E-05
	Nucleobase-containing compound metabolic process	3532	309	245.41	+	1.71E-03
	mRNA splicing, via spliceosome	368	46	25.57	+	2.64E-02
	Protein metabolic process	2807	244	195.04	+	2.70E-02
	Cellular component biogenesis	114	20	7.92	+	3.73E-02
	RNA splicing	272	36	18.9	+	4.72E-02
	RNA splicing, via transesterificationreactions	272	36	18.9	+	4.72E-02
	**Cellular component**					
	No significant over/under representation of genes					
	**Pathway**					
	Parkinson disease	88	18	6.11	+	1.22E-02

In comparison to controls, both the normozoospermic and asthenozoospermic groups had significant over-representation of the transcripts whose protein products localized to ribonucleoprotein complex, macromolecular complex and microtubule. Moreover, transcripts whose protein products localized to protein complex were significantly over-represented in normozoospermics; however, transcripts whose protein products localized to ribosome and cytosol were significantly over-represented in asthenozoospermics. Pathway analysis showed no over-representation in any of these comparisons (Table 2).

In comparison between the asthenozoospermic and normozoospermic groups, transcripts having molecular function in RNA binding, nucleic acid binding and structural constitution of ribosome showed significant over-representation. This comparison also showed significant over-representation of transcripts participating in biological processes such as translation, RNA splicing, mRNA splicing, and protein metabolic process. Further, in pathway analysis, significant over-representation of transcripts involved in Parkinson’s disease was observed (Table 2).

## Discussion

In this study, we identified a total of two thousand and eighty one differentially expressed transcripts. Gene ontology analysis of these transcripts revealed heterogeneous RNA population in spermatozoa. These transcripts included a large number of coding and non-coding RNAs. Gene set analysis of these transcripts revealed their roles in various metabolic pathways such as actin polymerization and depolymerization and its regulation, protein import into nucleus, glycolysis, and regulation of MAP kinase activity, regulation of cell motility, cell maturation, and regulation of secretion. We identified that a number of ribosomal protein transcripts (part of small and large ribosomes) were differentially expressed in both the asthenozoospermic and normozoospermic groups in comparison to the control group. Presence of the ribosomal protein transcripts in spermatozoa and their differential expression in asthenozoospermic and normozoospermic patients indicated a crucial role of ribosomes in the production of healthy sperm. Defects in ribosome biogenesis have already been associated with many diseases [20,21]. Recently, Gibbons et al, (2014) revealed that ribosomal DNA copy number is coupled with gene expression variation and mitochondrial DNA abundance in humans [22]. Mitochondria in sperm are the main source of energy and have their own translational machinery. Differential expression of the ribosomal proteins could affect assembly of ribosomes in mitochondria, which further could affect functions of mitochondria in spermatozoa. We observed no down-regulation of the ribosomal proteins in sperm of the normozoospermic patients in comparison to control individuals. In contrary to this, a number of ribosomal protein transcripts were down-regulated in sperm of asthenozoospermic patients in comparison to control individuals. This may be an indication of disrupted function of mitochondria in the asthenozoospermic patients. Further, Gur and Breitbart (2006) reported that nuclear encoded-proteins in mammalian sperm are translated by mitochondrial-type ribosomes and are expressed during their residence in the female reproductive tract until fertilization [23]. In this context, any possible dysfunction of ribosomes could compromise the fertilization process.

Among various differentially expressed transcripts, we identified the down-regulation of *HSPA4* in the asthenozoospermic group when compared with the normozoospermic infertile group. Held et al, (2006) have shown fertility defects in Hspa4l^-/-^ deficient mice where testis contained all stages of germ cells, but the number of mature sperm in epididymis and sperm motility were drastically reduced [24]. We observed the down-regulation of *HSP90AB1* transcript in the asthenozoospermic group in comparison to the control group, while this transcript was up-regulated in the normozoospermic group when compared with the control group. Moreover, we observed the down-regulation of *DAD1* (defense against cell death) gene in both the normozoospermic and asthenozoospermic groups in comparison to controls. An earlier study using Mouse Gene Expression microarray I (GEM1) in male mice had revealed that heat exposure could reduce mRNA levels of the *DAD1* gene in heat-shocked testis [25]. Thus, comparative analysis of differentially expressed heat shock and apoptosis genes indicated crucial roles of these genes in male fertility. We observed differential expression of a number of transcripts, such as *PRM1* [26], *SPATA18* [27], *TNP1* [28], *EIF4G2* [29], *CANX* [30], *RANBP9* [31], *TCP11* [32,33], which have previously been associated with male infertility. Ravel et al (2007) revealed that mutations in the protamine gene 1 are associated with male infertility [26]. Interestingly, we observed reduced protamine 1 (*PRM 1*) levels in the asthenozoospermic patients in comparison to the normozoospermic patients. Depa-Martynów et al, (2007) revealed that *PRM1* and *PRM2* mRNA levels are directly associated with fertilization and embryo development [34]. The authors observed reduced *PRM1* and *PRM2* mRNA levels in spermatozoa obtained from the patients in which *in vitro* fertilization (IVF) had failed. Further, we observed the down-regulation of *SPATA18* (spermatogenesis-associated 18 homolog) in the asthenozoospermic patients in comparison to the normozoospermic patients. Bornstein et al, (2011) revealed that *SPATA18* is a potential target for p53, which functions not only to suppress tumorigenesis, but also to maintain normal development and homeostatis [27]. *Spetex 1* (*SPATA18* homolog in rat) protein was found to localize at satellite fibrils associated with the outer dense fibers in the middle piece of sperm flagella [35]. Down-regulation of *SPATA18* in the asthenozoospermic group may suggest important function of this gene in sperm motility and fertility. Further, the genes encoding transition nuclear proteins (*TNP1* and 2) are crucial for production of healthy sperm. Transition nuclear proteins have a major role in chromosomal packaging during spermiogenesis. Miyagawa et al, (2013) have shown that deletion in the promoter region of the *TNP1* gene reduces its expression that can lead to male infertility [28]. We observed low expression of the *TNP1* transcript in the asthenozoospermic patients in comparison to the normozoospermic patients, which indicated its crucial role in sperm motility. Eukaryotic translation initiation factor (*EIF4G*) has been shown to be crucial in meiotic cell cycle progression and spermatic differentiation in Drosophila male germ cells [29]. *EIF4G2* mutant germ cells fail to form compact mitochondrial derivative and full elongation to spermatids. Interestingly, *EIF4G2* transcript was down-regulated in the asthenozoospermic group, but up-regulated in the normozoospermic group when each group was compared with the control group. Moreover, we observed down-regulation of *RANBP9* transcript in the asthenozoospermic group in comparison to the normozoospermic infertile group. Recently, Bao et al, (2014) have revealed that the *RANBP9* gene is involved in alternative splicing in the spermatogenic cells and is required for correct processing of mRNAs of many genes involved in sperm production [31]. In the absence of Ranbp9, many of *RANBP9* targeted and non-targeted mRNAs either showed aberrant splicing patterns or were dysregulated. The above evidence based analysis of gene expression data shows that transcriptome analysis has the potential of identifying suitable marker(s) for fertility evaluation.

Further, we identified differential expression of a number of transcripts related to reproduction and development. A few transcripts were specific to testis (*TCP11*, *TESK1*, *TSPYL1*, *ADAD1*) and a few originated from the Y-chromosome (*DAZ*, *TSPYL1*). A number of these transcripts have been linked to fertility and infertility in various studies on animals/humans. However, detailed investigations would be required to find out their role in spermatogenesis/male fertility. Earlier studies have reported the presence of silencing RNAs in spermatozoa [36–38], which could play roles as regulatory elements during chromosomal repackaging in spermatozoa and after fertilization in oocyte. We observed that in comparison to the control and normozoospermic groups, the asthenozoospermic group showed down-regulation of *PIWIL1* transcript; the latter encodes a member of the PIWI subfamily of argonaute proteins that are important to spermatogenesis and fertility. The members of this family are evolutionarily conserved proteins containing both the PAZ and Piwi motifs and play important roles in RNA silencing [39,40]. Our observation of differential expression of a number of transcripts important to various biological processes including reproduction and development strengthens the evidence that sperm RNA may participate in male fertility and some of these transcripts may serve as markers for male infertility.

In summary, this microarray study revealed the presence of a complex RNA population in spermatozoa. This RNA population included many coding and non-coding RNAs. A number of transcripts that participate in a host of cellular processes were differentially expressed between fertile and infertile individuals. Individual to individual variations in transcript levels were ruled out by pooling of sperm before RNA isolation. Gene ontology analysis revealed the presence of transcripts from different categories of biological processes including reproduction and development. What remains to be investigated is if these transcripts are mere representatives of past gene activity or they have to play active roles during further development post-fertilization. Nevertheless, it is largely clear that a number of transcripts have potential as infertility markers. Though this is the first such kind of study on Indian men and store transcripts could vary with geography and ethnicity, a large number of studies are further required for evaluation of regional and ethnic variations, if any. Transcript analysis, if established, can be a quick and easy method for fertility evaluation, and establishment of the role of RNAs in sperm fertility has the potential of identifying targets for infertility treatment and/or contraception. Nevertheless, further studies could explore differentially expressed transcripts for their role in spermatogenesis and male fertility.

## Supporting Information

S1 Fig Normalization of microarray raw data. MA and RMA (Robust multi-array average) plots showing the normalized data after background correction, log_2_ transformation and summarization.
(TIF)

S2 Fig Quality control graphs of microarray experiments. AP 1 and 2 represent asthenozoospermic replicates 1 and 2; CM 1 and 2 represent fertile control replicates 1 and 2; NP 1 and 2 represent normozoospermic infertile replicates 1 and 2.
(TIF)

S3 Fig Three group comparison and gene selection analysis. Heat map showing differentially expressed gene probesets for comparison among fertile control, asthenozoospermic infertile, and normozoospermic infertile groups.
(TIF)

S4 Fig Three group comparison and gene selection analysis. Box plot showing top four ranked differentially expressed gene probesets for comparison among fertile control, asthenozoospermic infertile, and normozoospermic infertile groups.
(TIF)

S5 Fig Three group comparison and gene set analysis. Heat map showing the variations in pathways taking into account the gene expressions involved in that pathway among normal fertile control, asthenozoospermic infertile, and idiopathic normozoospermic infertile groups.
(TIF)

S6 Fig Three group comparison and gene set analysis. Box plot showing the top four ranked differentially expressed pathways for comparison among normal fertile control, asthenozoospermic infertile, and idiopathic normozoospermic infertile groups.
(TIF)

S1 Table Gene selection analysis. Supplementary data on gene selection analysis when three groups were compared together (feature parameter set = 5000).
(XLSX)

S2 Table Gene set analysis. Supplementary data on gene set analysis when three groups were compared together (feature parameter set = 5000).
(XLSX)

S3 Table Co-expression gene network analysis. Supplementary data on co-expression gene network analysis when three groups were compared together (feature parameter set = 5000).
(XLSX)

S4 Table Gene selection analysis. Supplementary data on gene selection analysis when the three groups were compared with each other (feature parameter set = 5000).
(XLSX)
