# Supplementary figures and images for: Differential Genes Expression between Fertile and Infertile Spermatozoa Revealed by Transcriptome Analysis
Source: PLoS One. 2015 May 14;10(5):e0127007. doi: 10.1371/journal.pone.0127007 (PMC4431685; doi:10.1371/journal.pone.0127007)

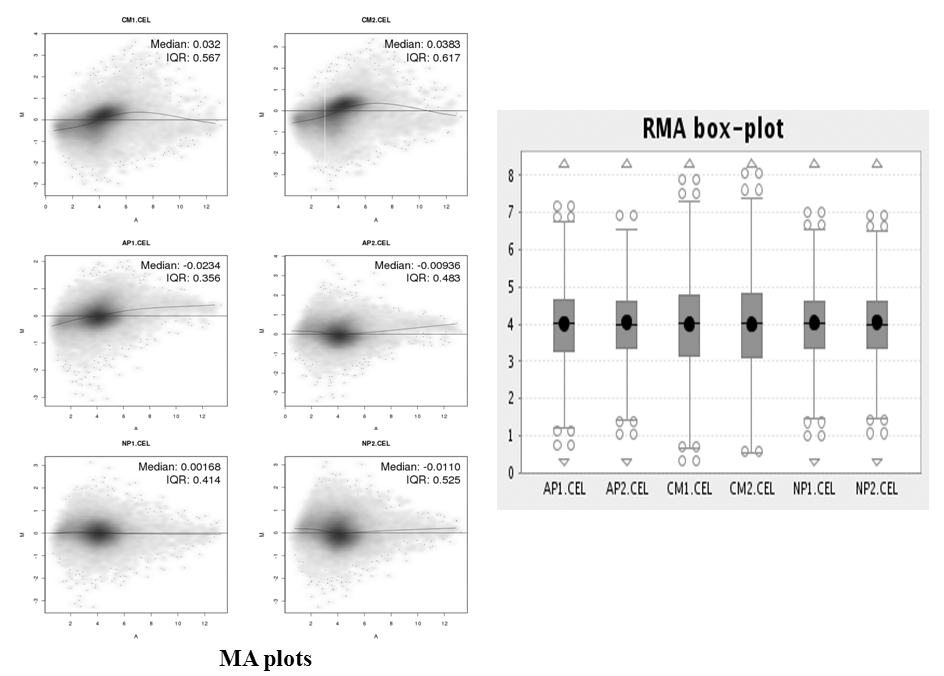

Supplement: S1 Fig — MA and RMA (Robust multi-array average) plots showing the normalized data after background correction, log2 transformation and summarization. (TIF) [file pone.0127007.s001.tif]

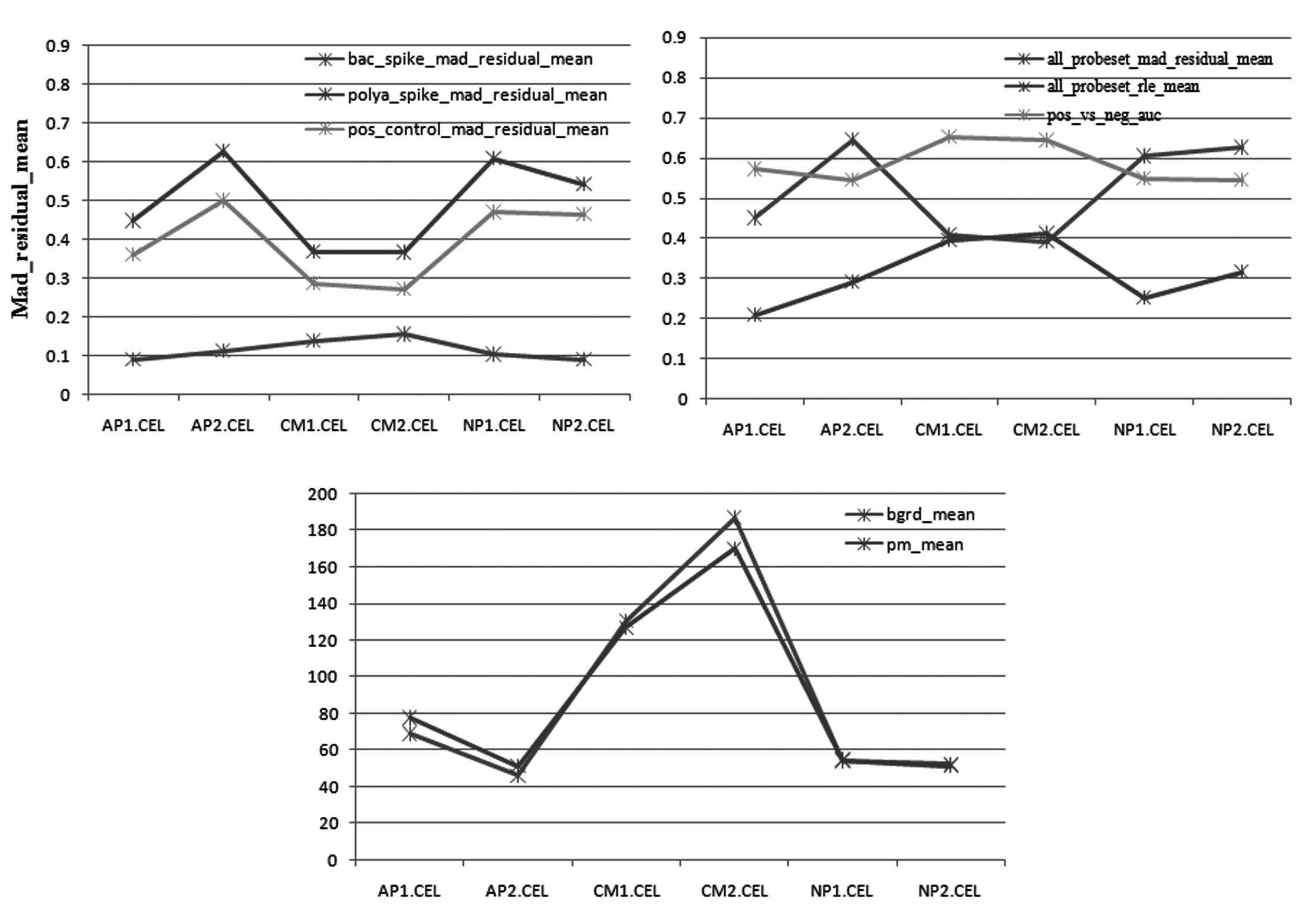

Supplement: S2 Fig — AP 1 and 2 represent asthenozoospermic replicates 1 and 2; CM 1 and 2 represent fertile control replicates 1 and 2; NP 1 and 2 represent normozoospermic infertile replicates 1 and 2. (TIF) [file pone.0127007.s002.tif]

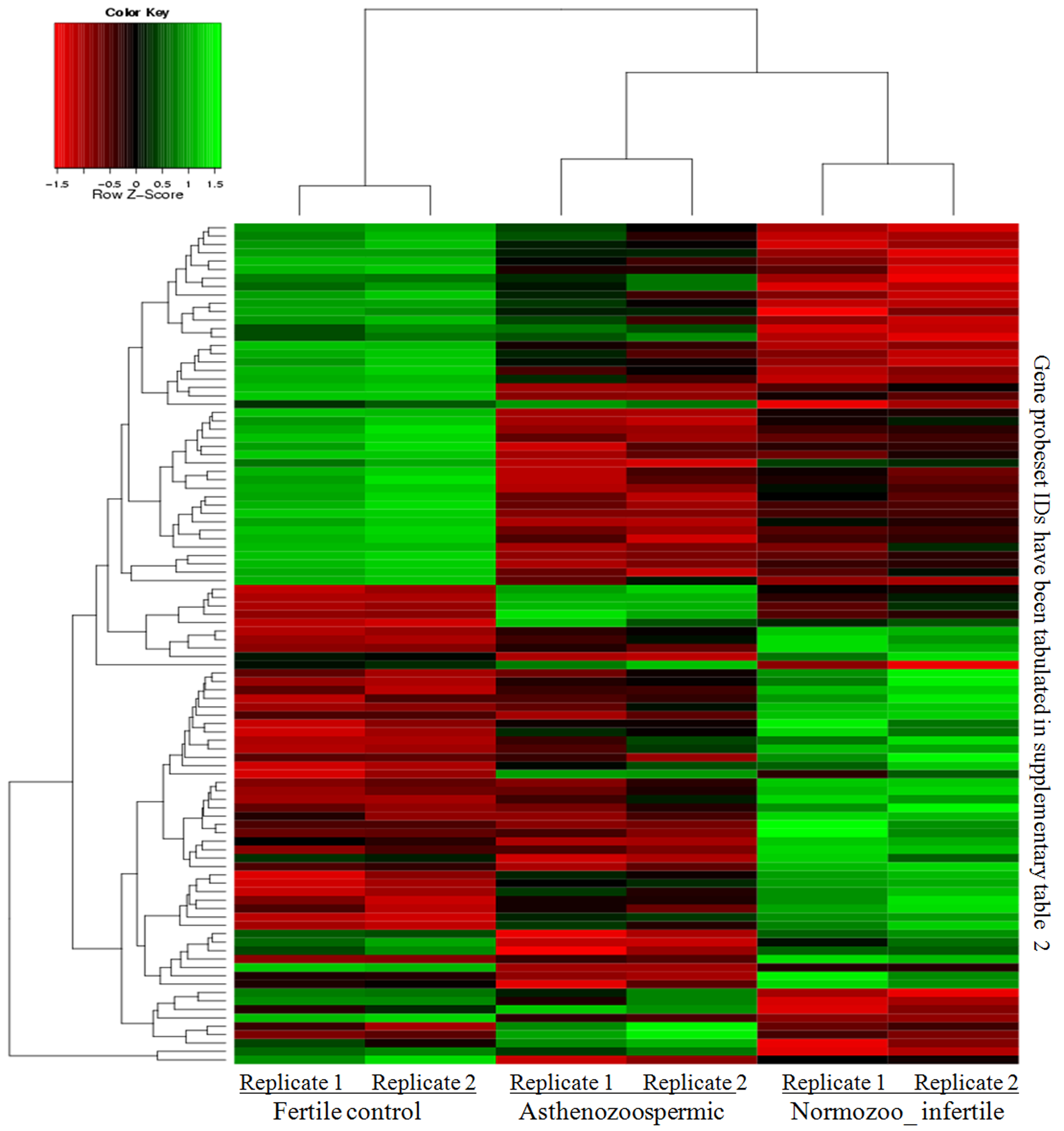

Supplement: S3 Fig — Heat map showing differentially expressed gene probesets for comparison among fertile control, asthenozoospermic infertile, and normozoospermic infertile groups. (TIF) [file pone.0127007.s003.tif]

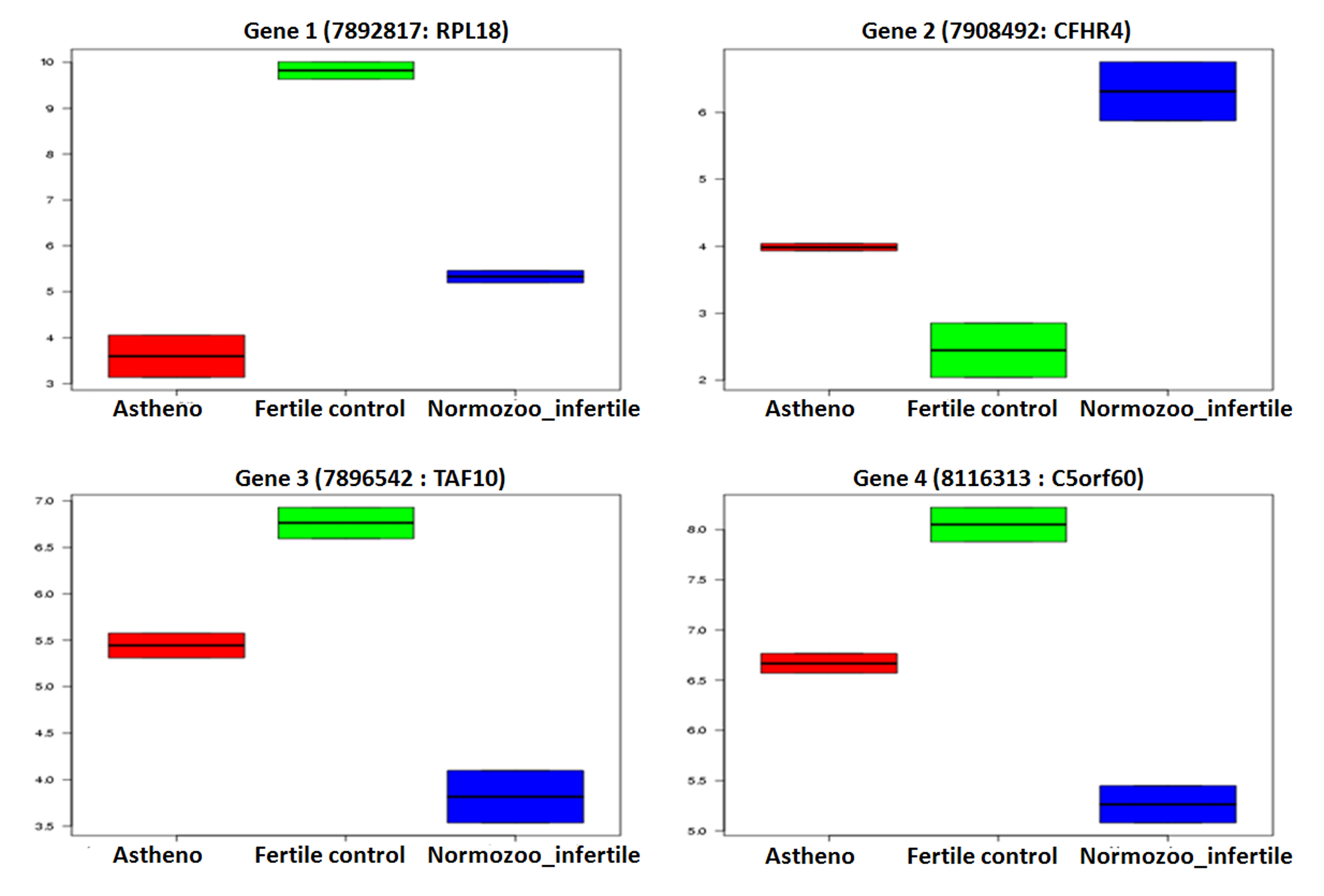

Supplement: S4 Fig — Box plot showing top four ranked differentially expressed gene probesets for comparison among fertile control, asthenozoospermic infertile, and normozoospermic infertile groups. (TIF) [file pone.0127007.s004.tif]

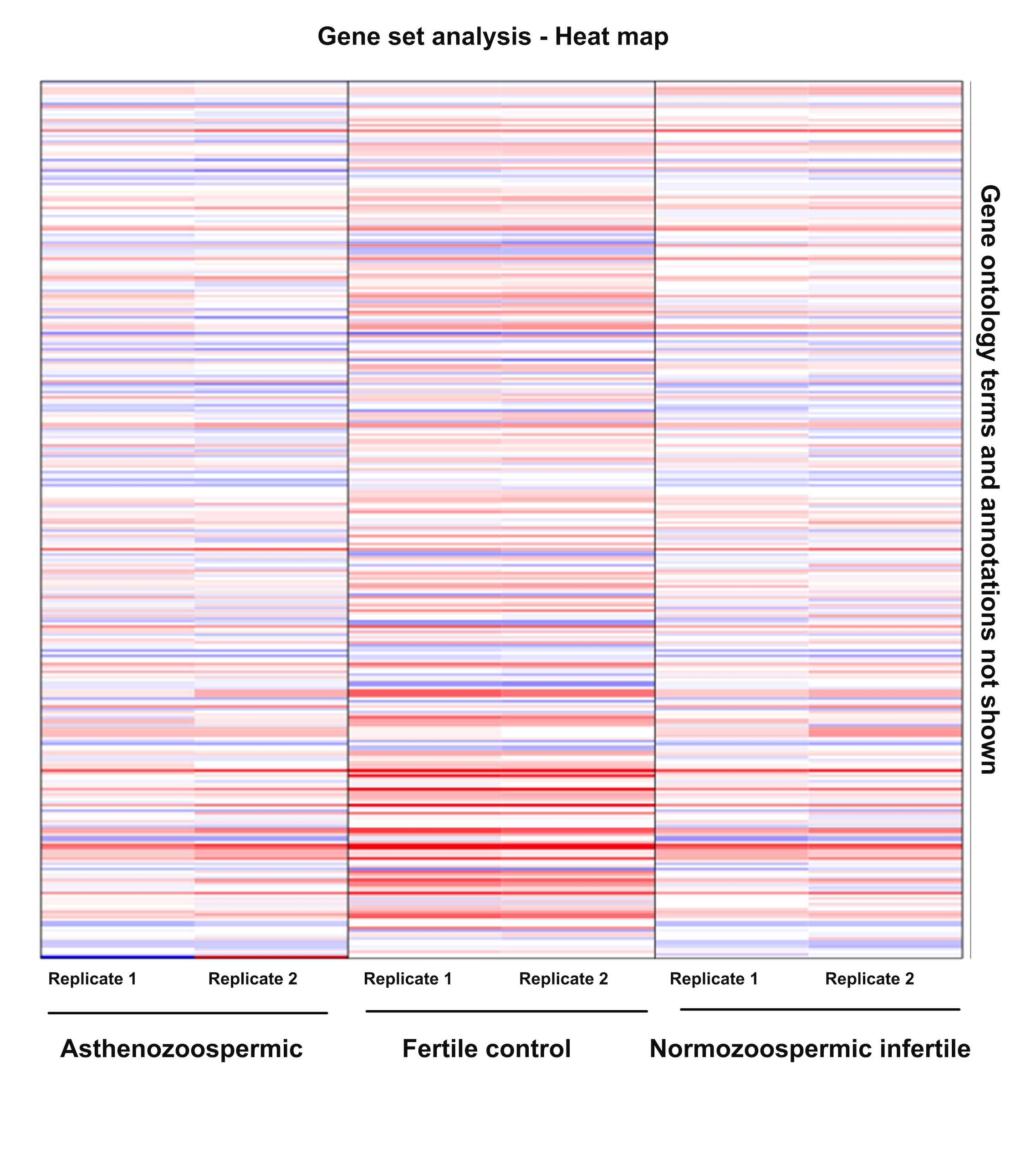

Supplement: S5 Fig — Heat map showing the variations in pathways taking into account the gene expressions involved in that pathway among normal fertile control, asthenozoospermic infertile, and idiopathic normozoospermic infertile groups. (TIF) [file pone.0127007.s005.tif]

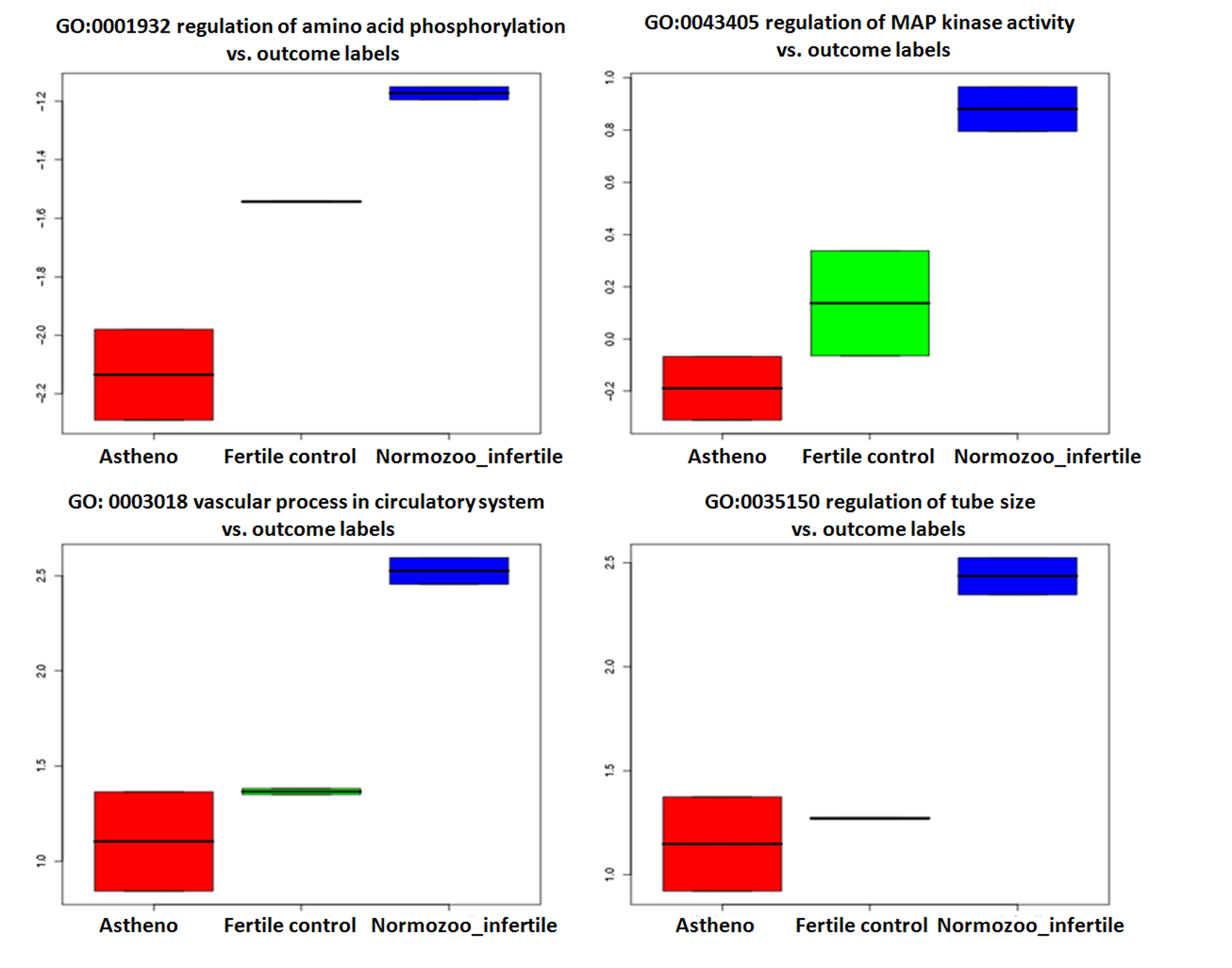

Supplement: S6 Fig — Box plot showing the top four ranked differentially expressed pathways for comparison among normal fertile control, asthenozoospermic infertile, and idiopathic normozoospermic infertile groups. (TIF) [file pone.0127007.s006.tif]
